# Subtle Changes in Myocardial Work Indices Assessed by 2D-Speckle Tracking Echocardiography Are Linked with Pathological LV Remodeling and MACEs Following an Acute Myocardial Infarction Treated by Primary Percutaneous Coronary Intervention

**DOI:** 10.3390/diagnostics13193108

**Published:** 2023-09-30

**Authors:** Diana-Aurora Arnautu, Alexandru Gheorghiu, Sergiu-Florin Arnautu, Mirela-Cleopatra Tomescu, Claudiu-Daniel Malita, Christian Banciu, Cristina Vacarescu, Ioana Ionac, Silvia Luca, Dragos Cozma, Cristian Mornos, Dan Gaita, Constantin-Tudor Luca

**Affiliations:** 1 Multidisciplinary Heart Research Center, “Victor Babes” University of Medicine and Pharmacy, 2 Eftimie Murgu Sq., 300041 Timisoara, Romania; aurora.bordejevic@umft.ro (D.-A.A.); dralexandreugheorghiu@gmail.com (A.G.); tomescu.mirela@umft.ro (M.-C.T.); 2 Department of Internal Medicine, ”Victor Babes” University of Medicine and Pharmacy, 2 Eftimie Murgu Sq., 300041 Timisoara, Romania; arnautu.sergiu@umft.ro (S.-F.A.); banciu.christian@umft.ro (C.B.); 3 Institute of Cardiovascular Diseases Timisoara, 13A Gheorghe Adam Street, 300310 Timisoara, Romania; vacarescucristina@yahoo.com (C.V.); ioana_ionac@yahoo.com (I.I.); silvia.luca0@yahoo.com (S.L.); dragoscozma@gmail.com (D.C.); mornoscr@yahoo.com (C.M.); dgaita@cardiologie.ro (D.G.); costiluca67@yahoo.com (C.-T.L.); 4 Timisoara Municipal Clinical Emergency Hospital, 12 Revolution of 1989 Bd., 300040 Timisoara, Romania; 5 Department of Radiology and Medical Imaging, ”Victor Babes” University of Medicine and Pharmacy, 2 Eftimie Murgu Sq., 300041 Timisoara, Romania; 6 Department of Cardiology, ”Victor Babes” University of Medicine and Pharmacy, 2 Eftimie Murgu Sq., 300041 Timisoara, Romania; 7 Research Center of the Institute of Cardiovascular Diseases Timisoara, 13A Gheorghe Adam Street, 300310 Timisoara, Romania

**Keywords:** acute myocardial infarction, myocardial work indices, left ventricular remodeling, outcome

## Abstract

The goal of this study was to assess whether subtle changes in myocardial work indices may predict left ventricular (LV) remodeling and major cardiac events (MACEs) in patients with a first ST-elevation acute myocardial infarction (STEMI) and preserved LVEF after successful myocardial revascularization with PCI. Methods. Consecutive STEMI patients in sinus rhythm and with an LV ejection fraction ≥ 50% following a successful PCI were recruited. Conventional and two-dimensional speckle tracking echocardiography (2D-STE) was conducted within 36 h of the PCI and 3 months later. Patients having an increase of more than 20% in LV diastolic volume were included in the LV remodeling group. MACEs were noted throughout a four-year period of follow-up. Results: The study comprised 246 STEMI patients with a mean age of 66; 72% of whom were men. In 24% (58) of the patients, LV remodeling developed. These patients were older, more frequently hypertensive, and had a smoking history. They also exhibited significantly lower baseline and 3-month values for the myocardial global index (GWI), global constructive work (GCW), and global myocardial efficiency (GWE). The cut-off values of 1670 mmHg% for GWI and 83% for GWE were predictive of LV remodeling (*p* < 0.0001). During the four-year follow-up period, 19% of STEMI patients experienced a MACE, involving 15% from non-LV remodelers and 34% from LV remodelers (*p* = 0.01). The cut-off values for baseline GWI of 1680 mmHg% and baseline GWE of 84% had the best accuracy in predicting MACEs. In conclusion, non-invasive myocardial work indices offered a reproducible and accurate method to predict post-MI LV remodeling and MACEs.

## 1. Introduction

After an acute myocardial infarction (AMI) treated with primary percutaneous coronary intervention (PCI), left ventricular (LV) remodeling is a key predictor of both short-term and long-term major cardiac adverse events (MACEs) [1]. It is characterized by changes in ventricular architecture that impact both infarcted and non-infarcted segments, followed by consistent growth in LV diastolic and systolic volumes [2], and it has been associated with arrhythmias, heart failure (HF), and higher death risk. Despite the fact that primary PCI has significantly enhanced the prognosis of patients with AMI, harmful remodeling of the left ventricle still appears in approximately 30% of cases [3]. It is essential to assess the risk of all AMI patients to accurately expose them to therapy that may enhance the prognosis.

Conventional echocardiography is the recommended method for identifying HF in patients with AMI by assessing the LV ejection fraction (EF) and the wall motion score index [4]. LV strain and strain rate determined by two-dimensional speckle tracking echocardiography (2D-STE) are newer, more accurate techniques for assessing myocardial performance, with the ability to discover discrete abnormalities in LV performance, particularly among subjects with AMI and preserved LVEF [5,6].

Myocardial work (MW) imaging employing 2D-STE is a sensitive tool for detecting localized wall motion abnormalities as well as a strong method for measuring myocardial performance beyond LVEF [6,7]. It assesses myocardial distortion by interpreting strain in conjunction with a dynamical noninvasive LV systolic pressure evaluation and is not impacted by LV systolic pressure [8,9]. Recent studies revealed that decreased MW indices might recognize persons with acute myocardial injury with excellent accuracy [10,11].

The objective of this research was to assess whether subtle abnormalities of myocardial work indices may predict LV remodeling and MACEs in subjects with a first acute ST-elevation myocardial infarction (STEMI) and preserved LVEF after efficient myocardial revascularization with PCI.

## 2. Materials and Methods

### 2.1. Patient Selection

This is a case-control prospective research that included consecutive patients admitted with a first STEMI between January 2018 and May 2019, in sinus rhythm and with an LVEF ≥ 50% after an efficient primary PCI performed within 12 h of symptoms beginning.

Patients with unsatisfactory echocardiographic images, previous myocardial infarction, cardiac pacemaker/defibrillator, open-chest surgery, moderate or severe mitral regurgitation, moderate or severe aortic stenosis, severe chronic renal failure, liver disease, and non-cardiac diseases with a life expectation below a year, have been excluded from the research.

The study was performed at the Cardiology Departments of the Clinical Emergency Municipal Hospital and the Institute of Cardiovascular Disease in Timisoara. Conventional and 2D-STE were carried out at baseline (within 36 h following the PCI) and at 3 months after the event.

Corresponding to the LV diastolic volume (DV) at baseline and at 3 months following the STEMI, the patients were split into two groups: group A (without LV remodeling) and group B (with LV remodeling). LV remodeling was described as an increase of ≥20% of LVDV from enrollment [2].

The patients were monitored for a period of 4 years and the following endpoints were recorded as MACEs: cardiovascular mortality, myocardial revascularization by PCI or aorto-coronary by-pass grafting (CABG), and hospitalizations for heart failure.

The Ethics Commission of Timisoara’s “Victor Babes” University of Medicine and Pharmacy agreed with the research (nr 36/2017). According to the Helsinki Declaration, all patients completed an informed consent form to participate in the research.

### 2.2. Data Extraction

Data regarding sex, age, heart disease risk factors (hypertension, hypercholesterolemia, diabetes mellitus, obesity, history of smoking), clinical exam information, laboratory data, 12 leads—resting electrocardiogram, 2D-transthoracic echocardiography, and coronary angiography results were obtained from the patients’ records at admission for the STEMI.

### 2.3. Definition of Covariates

STEMI was confirmed if at least two of the subsequent conditions occurred: (1) typical angina exceeding 20 min; (2) ST-segment elevation of ≥1 mV continuing for more than 0.08 s in at least two adjacent leads; (3) transitory enhancement in cardiac enzymes to more than twofold the normal level [12].

The STEMI patients were assigned to a Killip class based on their clinical presentation: no heart failure, class 1; moderate heart failure, class 2; acute pulmonary edema, severe heart failure, class 3; and cardiogenic shock, class 4 [13].

### 2.4. Blood Tests

At admission, peripheral plasma probes were obtained. A Modular Analytics E170 NT-proBNP immunoassay (Roche Diagnostics, Mannheim, Germany) was used. Standard biochemical tests were performed throughout the hospital stay, including hemoglobin, glycated hemoglobin, creatinine, potassium, sodium, LDL cholesterol, and creatin-phosphokinase MB. To determine the predicted rate of glomerular filtration, the Modification of Diet in Renal Disease (MDRD) formula was utilized [14].

### 2.5. PCI

The PCI was accomplished in accordance with the acknowledged techniques after the STEMI was confirmed during the first 12 h from the beginning of symptoms. The standard procedure for treating the culprit lesion involved using a balloon for pre-dilatation and insertion of drug-eluting stents. Significant stenosis was defined as more than 75% in the circumflex, right, and anterior descending coronary arteries or more than 50% in the left main coronary trunk. Multivessel coronary artery disease (CAD) was diagnosed when significant stenosis was found in more than one coronary artery. A PCI was determined to be successful when a TIMI flow 3 was obtained [15].

### 2.6. Echocardiography

Within 36 h of the PCI, a baseline echocardiogram was performed in the hospital utilizing a GE Vivid E7 ultrasound system (GE Health Medical, Milwaukee, WI, USA). A second echocardiography test was performed three months following the first one.

#### 2.6.1. Conventional Echocardiography

Simpson’s biplane approach, as endorsed by the American Society of Echocardiography [16] was used to calculate LV volumes and LVEF. To visually assess regional wall motion, a 17-segment representation was utilized, with every segment characterized as 1, normal; 2, hypokinesia; 3, akinesia; and 4, dyskinesia [16]. By averaging the segmental values, we obtained the wall motion score index. To evaluate the LV function during diastole, the following parameters were determined: the maximal protodiastolic velocity of the transmitral flow (peak E), the maximal telediastolic velocity (peak A), the E/A ratio, and the isovolumic relaxation time (IVRT) [16,17].

#### 2.6.2. D-STE

The 2D-STE method was applied to evaluate LV distortion models [18]. The Echo PAC system version 204 (GE Vingmed) was used for offline analysis. In the 4-, 3-, and 2-chamber apical incidences, the global longitudinal strain and its strain rate were recorded. Although the program identified the myocardium spontaneously, the myocardial boundary was manually corrected by the investigator to improve efficiency. In addition, each short-axis and apical image was split into 6 regions, resulting in 18 divisions. The global peak longitudinal strain (GLS) was calculated as the average of the 18 segments.

#### 2.6.3. Myocardial Work Determinations

We stated the systolic blood pressure value and developed non-invasive myocardial work indices, which are the product of segmental shortening velocity and momentary LV pressure. The entire LV work during systole is determined by the global myocardial work index (GWI). Myocardial constructive work (GCW) estimates all the work contributing to pump function (systolic shortening in addition to isovolumic relaxation). Global wasted work (GWW) estimates the amount of work that does not provide ejection (systolic myocardial lengthening in addition to any shortening for the duration of isovolumic relaxation). The ratio of GCW to the total of GCW plus GWW is used to determine global work efficiency (GWE). The pressure-strain loop diagram is utilized for determining global MW, whereas the GCW and GWW parameters are determined from the contraction pattern of regional segments [19,20].

**Figure 1:**
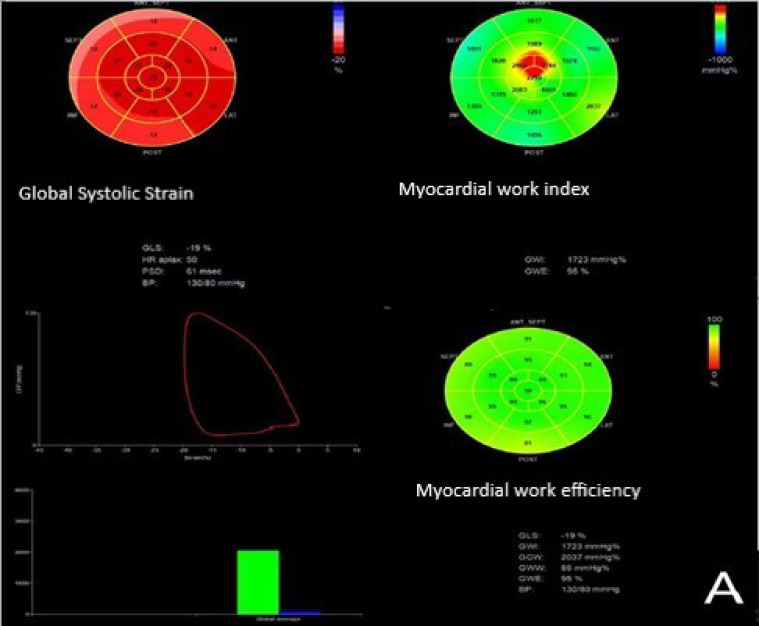

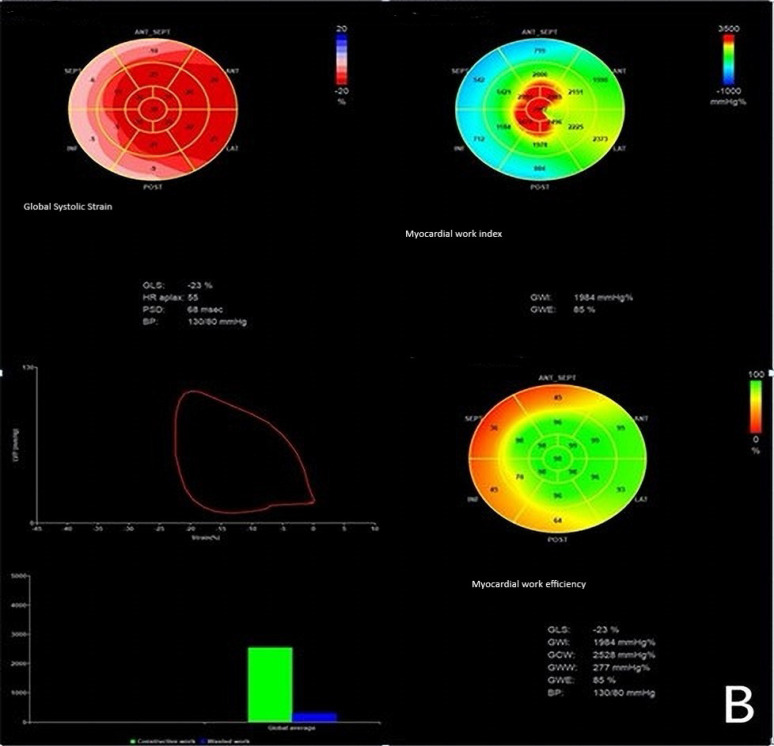
Bull’s eye of baseline GLS, GWI, and GWE in a STEMI patient without (**A**) and with (**B**) LV remodeling. In the global longitudinal strain Bull’s eye, the bright red color denotes a normal range in strain values (<−16%), light red denotes reduced value (−16 to −11%), light pink (−10 to −6%) and pale pink (−5 to 0%) denote severely reduced values, and blue denotes a positive value suggesting paradoxical systolic expansion. In the myocardial work index Bull’s eye, the green color represents the constructive myocardial work, while the blue color represents the waisted myocardial work. Abbreviations: GLS, global longitudinal strain; GWI, global work index; GWE, global work efficiency; STEMI, ST-segment elevation acute myocardial infarction; LV, left ventricular.

Figure 1 shows baseline myocardial work indices in a STEMI patient without (A) and with (B) LV remodeling.

### 2.7. Statistical Analysis

Categorical variables were represented using numbers and percentages. According to Kolmogorov-Smirnov tests, continuous variables normally distributed were presented as means with a standard deviation of 1, whereas those non-normally distributed were given as median (25th, 75th percentile).

For continuous and regularly distributed values, the paired t-test was used to compare the subject groups. The Mann-Whitney U-test was used to compare continuous non-normally distributed variables, and the chi-squared test was used to compare categorical variables. The odds ratio (OR) and confidence interval (CI) of many factors associated with LV remodeling and MACEs were determined using univariate analysis. The univariately significant factors linked to LV remodeling were included in a multivariate analysis, using a backward stepwise logistic regression model. The independent variables identified by multivariate logistical regression were analyzed using the receiver operating characteristic (ROC) to determine their accuracy. For survival analyses, the Kaplan-Meier procedure was utilized. We utilized the MedCalc Statistical Software version 22.003 (MedCalc Software Ltd., Ostend, Belgium) for statistical analysis. Two-tailed *p* < 0.05 values were regarded as statistically notable.

## 3. Results

### 3.1. Clinical Characteristics of the STEMI Patients

Seventeen of the 263 STEMI patients that were enrolled at start-up were excluded and not analyzed (11 were lost due to poor echocardiographic image quality, 5 due to atrial fibrillation at the 3-month echocardiography, and 1 due to PCI repeated before the 3-month evaluation). Finally, 246 patients aged 31 to 79 years (mean age 66 ± 13 years) were enrolled in the research, including 178 (72%) men.

At the 3-month echocardiographic evaluation, we found that 58 (24%) of the patients had LV remodeling (Table 1). The study found that patients with LV remodeling were generally older, with a mean age of 72 compared to 65 years for those without LV remodeling. Additionally, these patients were more likely to have a history of hypertension and smoking and had higher peak creatine MB-kinase levels. They also exhibited Killip classes 3 and 4 more often. According to baseline angiography, they were more likely to have multi-vessel coronary artery disease. All the patients who were part of the study had TIMI flow 3 in the culprit lesion vessel and their LVEF was over 50% after undergoing primary PCI. There were no significant differences in discharge medications between the two groups.

### 3.2. Angiographic Results

The study reveals that there were no significant disparities among the groups with respect to the localization of the culprit’s vessel. However, the LV remodeling group exhibited a higher proportion of multi-vessel coronary artery disease. Furthermore, it was observed that the initial patency of the infarct-related artery was present in 44 (18%) of the STEMI patients, with a greater frequency in the non-remodelers in comparison to the remodelers, although this contrast was not deemed statistically substantial (*p* = 0.16).

### 3.3. Echocardiographic Data

The echocardiographic data are shown in Table 2. At baseline, the patients with LV remodeling had significantly lower GWI, GCW, and GWE (*p* < 0.0001) and higher GWW (*p* = 0.001). After 3 months, they presented significantly lower LVEF *p* < 0.0001), higher LVDV (*p* = 0.03), higher LV systolic volume (*p* = 0.01), and higher wall motion score index (*p* < 0.01). Their myocardial work indices revealed significantly lower GWI, GWE, and GCW and higher GWW (*p* < 0.01) compared to the non-remodelers.

### 3.4. Independent Predictors of 3-Months LV Remodeling

According to Table 3, utilizing a univariate analysis, the development of LV remodeling was significantly linked with age, hypertension, hypercholesterolemia, smoking status, Killip class, peak creatin-kinase MB level, two- and three-vessel CAD, baseline GWI, GCW, GWE, and GWW. Killip class, baseline GWI, and baseline GWE have been identified as independent predictors of LV remodeling by multivariate logistic regression.

The most powerful predictor of LV remodeling was baseline GWE (%), with an area under the curve (AUC) of 0.95, significantly higher than the AUC of the ROC curve for GWI (*p* < 0.04) and the AUC for the Killip class (*p* < 0.0001). The analysis of the ROC curves showed that the cut-off values predictive of LV remodeling were a baseline GWE ≤ 83% (sensitivity 89.7%, specificity 75.6%, *p* < 0.001), a baseline GWI ≤ 1670 mmHg% (sensitivity 82.7%, specificity 76,4%, *p* < 0.0001), and a Killip class > 2 (sensitivity 77.5%, specificity 72.1%, *p* < 0.0001).

**Figure 2:**
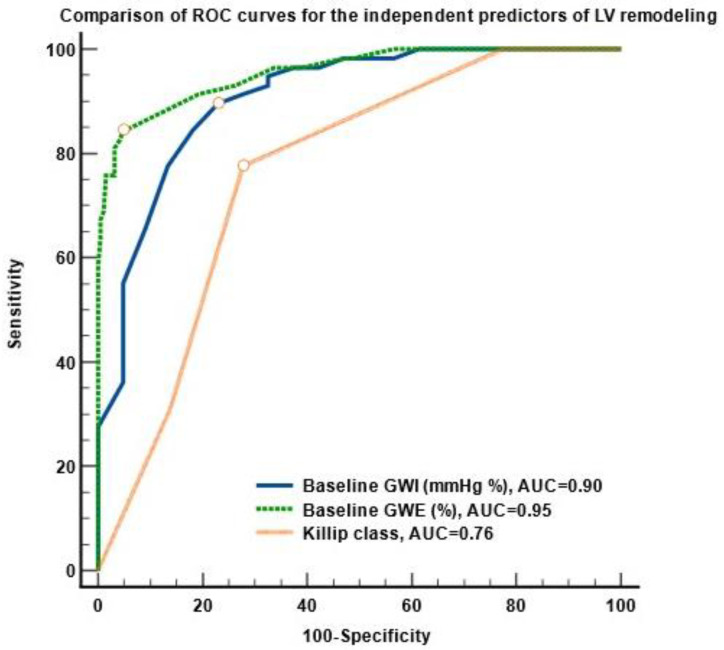
Comparison of the ROC curves of the independent predictors of LV remodeling in STEMI patients. Abbreviations: ROC, receiver operating curve, AUC, area under the curve, LV, left ventricular, ST-segment elevation acute myocardial infarction; GWI, global work index; GWE, global work efficiency.

Figure 2 illustrates the ROC curves for the independent predictors of LV remodeling.

### 3.5. Independent Predictors of MACEs

**Figure 3:**
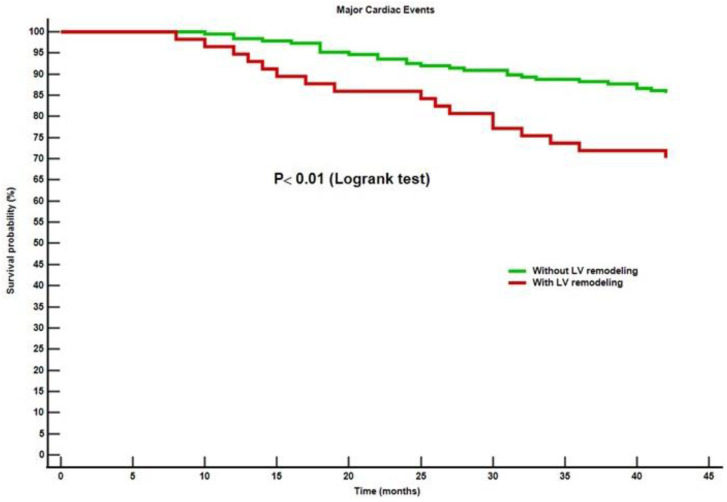
Kaplan-Meier survival curves without major cardiac events in STEMI patients with and without LV remodeling. Abbreviations: ST-segment elevation acute myocardial infarction; LV, left ventricular.

Throughout the 4-year follow-up period, 48 patients (19%) suffered a MACE, 28 (15%) from the non-remodelers and 20 (34%) from the LV-remodelers (*p* = 0.01). In addition, 29 (12%) patients were hospitalized for heart failure, and 15 (6%) suffered a new myocardial revascularization by a PCI. No patient died. The hospitalization rate was 9% in group A, and 19% in group B (*p* = 0.03). The myocardial revascularization rate was 4% in group A and 12% in group B (*p* = 0.02). The Kaplan-Meier survival curve for MACEs is presented in Figure 3 and shows a significantly higher survival time without a MACE in the STEMI patients without LV remodeling (*p* < 0.01).

MACEs were notably linked in univariate analysis to Killip class, three-vessel coronary artery disease, baseline values of NT-pro BNP, peak CK-MB, GWI, and CWI (Table 4). Utilizing a multivariate analysis, Killip class and baseline values of peak CK-MB, GWI, and CWI were identified as independent predictors for the risk of MACEs (Table 4).

**Figure 4:**
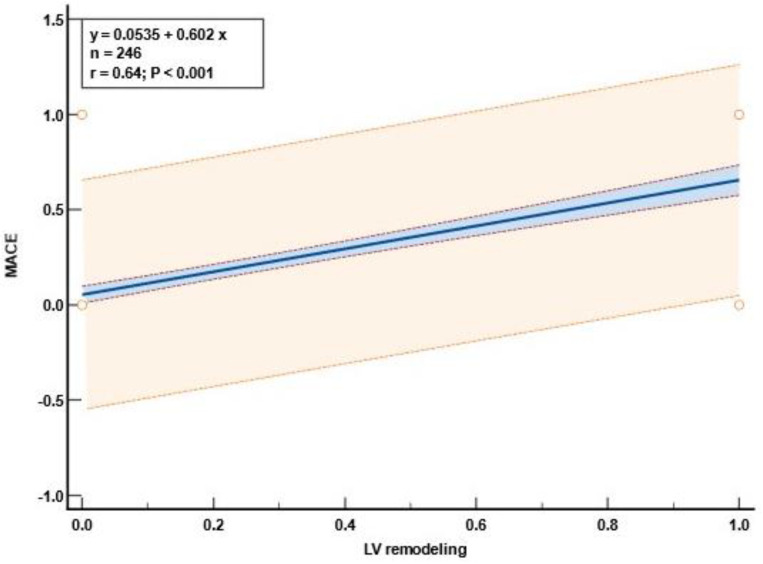
Significant positive correlation between MACEs and early LV remodeling. Abbreviations: MACEs, major cardiac events; LV, left ventricular.

The occurrence of a MACE was significantly associated with the presence of LV remodeling, as shown in Figure 4, *p* < 0.001.

**Figure 5:**
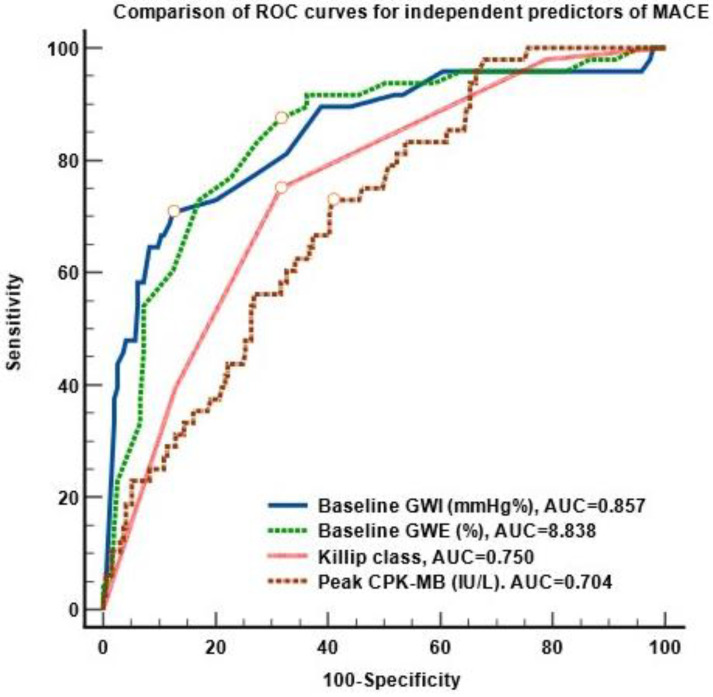
Comparison of the ROC curves of the independent predictors of MACEs in STEMI patients. Abbreviations: MACEs, major cardiac events; STEMI, ST-segment elevation acute myocardial infarction; ROC, receiver operating curve, AUC, area under the curve, GWE, global work efficiency; GWI, global work index, CPK-MB, creatine kinase MB- isoenzyme.

The ROC curves of the independent predictors of MACEs in STEMI patients with preserved LVEF after successful primary PCI are shown in Figure 5.

The most powerful predictors of MACEs were baseline GWE and baseline GWI, with no significant difference between the AUCs of their ROC curves (*p* > 0.05). We found no table differences when comparing these AUCs with the AUCs of the ROC curves for peak CK-MB (*p* < 0.01) and Killip class (*p* < 0.04).

Analyzing the ROC curves, we found the following cut-off values associated with the occurrence of MACEs: baseline GWI ≤ 1680 mmHg% (sensitivity 70.8%, specificity 87.8%, *p* < 0.001), baseline GWE ≤ 84% (sensitivity 87.5%, specificity 68%, *p* < 0.001), baseline CK-MB > 236 IU/L (sensitivity 72.9%, specificity 57.5%, *p* < 0.001) and Killip class > 2 (sensitivity 75%, specificity 69%, *p* < 0.0001).

## 4. Discussion

To our awareness, this research represents the first prospective study to evaluate the variations of MW indices in STEMI patients with preserved LVEF after a successful primary PCI and to correlate these variations with early (3 months) LV remodeling and the incidence of MACEs during a 4-year follow-up period.

All the study patients had a TIMI flow of 3 in the culprit lesion vessel, which is a good sign. Additionally, their LVEF was over 50% after the primary PCI. These parameters indicate that the treatment has been successful and that the included patients have a low risk of developing LV remodeling [21].

Several studies have revealed that a significant proportion, approximately 10–20%, of patients diagnosed with STEMI display spontaneous coronary recanalization before angioplasty [22]. This phenomenon is considered a favorable sign, as patients exhibiting such characteristics tend to have a better prognosis than those with persistent occlusion. These benefits include a smaller infarct size, lower incidence of heart failure, and improved early and late survival rates. The results of our study indicate that 18% of patients diagnosed with a first STEMI and an LVEF ≥ 50% demonstrated an initial patency of the infarct-related artery. A higher proportion of non-remodelers (20%) presented with initial patency compared to those who remodeled (12%), but this difference was not statistically significant. These findings provide valuable insights into the incidence of initial patency in STEMI patients and suggest further investigation is necessary to fully understand the underlying mechanisms and implications of these observations.

We considered it crucial to continue monitoring these patients to ensure continued improvement in their condition. This ongoing monitoring is essential to guarantee that they are receiving the best possible care and treatment and to make any necessary adjustments to their care plan to ensure a successful outcome.

The post-infarction remodeling of the left ventricle is a complex process that can have serious implications for patients. This remodeling typically involves an increase in both systolic and diastolic left ventricular volumes, which can indicate a poor prognosis for recovery. One common definition of this remodeling is an increase in end-diastolic left ventricular volume by 20% at 6 months after MI compared with the baseline, although early remodeling can be diagnosed as early as one month after the MI [3]. Our team chose to evaluate LV remodeling at an intermediate time of 3 months to better understand the progression of this process and its impact on patient outcomes.

Our study demonstrates that noninvasive myocardial work indices provide valuable data for the identification of STEMI patients with a risk of experiencing LV remodeling and major cardiac events. We found that the STEMI patients who developed early LV remodeling presented more severe impairment of all MW indices at baseline. The 3-month follow-up examination showed that, with the exception of GWW, all MW indices improved, but still remained significantly lower than in the patients without LV remodeling (*p* < 0.001).

MW is an efficient, quantifiable, and repeatable noninvasive imaging method that has demonstrated its superiority compared to left ventricular ejection fraction and global longitudinal strain in detecting patients with ischemic heart disease. In the healthy heart, the LV contraction is uniform and the work performed by the LV segments is efficient. In patients with ischemic heart disease, the myocardial contraction in the ischemic regions is not homogenous and the amount of wasted myocardial work is larger. In patients with AMI, the infarcted regions are stretched by the non-infarcted ones. The consequences are myocyte hypertrophy and elongation in the non-infarcted regions, leading to increased wall mass, increased LV volumes, increased wasted myocardial work, and decreased myocardial work efficiency [23,24].

A recent study including STEMI patients treated with PCI, with noninvasive myocardial work indices measured within 48 h after admission, found that a culprit vessel regional MWI ≤ 1129 mmHg% was significantly associated with early (3 months) LV remodeling [25]. Lustosa et al. [26], found that the prevalence of impaired myocardial work indices was higher in patients with LV remodeling compared to patients without. This was a retrospective study of 600 STEMI patients treated by PCI with myocardial work indices determined 3 months after the hospitalization for AMI.

In the current study, noninvasive myocardial work indices were measured within 36 h after the primary PCI in STEMI patients and after 3 months. We also found that the patients who developed early LV remodeling presented lower baseline values of myocardial work indices compared with their counterparts. These differences are probably explained by the larger ischemic area, and consequently, the more dispersed metabolic changes that can be successfully identified by myocardial work. During myocardial ischemia, the heart reduces the synthesis of adenosine triphosphate due to alterations in metabolism. Russell et al. demonstrated the association between MW indices and glucose metabolism by positron emission tomography using 18F-fluorodeoxyglucose [23].

At the 3-month evaluation, GWI, GCW, and GWE increased in both patient groups, but the values remained significantly lower in the patients who developed LV remodeling (*p* < 0.001). Our research findings suggest that cut-off values of ≤1670 mmHg% for GWI and ≤83% for GWE can accurately predict early (within 3 months) LV remodeling, with a significant *p*-value of <0.0001. These findings are crucial in identifying patients at risk for LV remodeling, enabling early intervention and better outcomes.

Low baseline GWI and GWE were also significant independent predictors for the occurrence of a MACE during the 4-year follow-up period. In our study, the cut-off values of a baseline GWI ≤ 680 mmHg%, and a baseline GWE ≤ 84% showed the highest accuracy in predicting long-term MACEs in STEMI patients. Based on the findings of Coisne et al.’s study, patients with STEMI and NSTEMI who have a GWE below 91% are at a higher risk of experiencing poor outcomes within 2 years of monitoring [27]. Interestingly, our research found a lower GWE threshold of 84%, which could be attributed to the differences in the timing of the analysis following AMI and the inclusion of NSTEMI patients in Coisne et al.’s study. Nonetheless, these findings highlight the importance of closely monitoring GWE in patients with AMI to improve clinical outcomes.

Evidence suggests that revascularization procedures offer great benefits in terms of improving survival rates in STEMI patients. However, not all myocardium can experience improvement through revascularization. Studies have shown that even after successful revascularization of infarct-related arteries, there is still a relatively high incidence of microvascular obstruction or coronary microvascular dysfunction [28]. To detect impaired microvascular perfusion in patients with STEMI after revascularization, there are several imaging modalities available, such as Single-Photon Emission CT (SPECT), dobutamine stress echo, Cardiac Magnetic Resonance Imaging (CMR), Positron Emission Tomography (PET) imaging with F18-fluorodeoxyglucose (FDG), Speckle-Tracking Echocardiography (STE), and Myocardial Contrast Echocardiography (MCE). It is important to accurately detect any impaired LV function in these patients to ensure proper treatment and care.

According to our results, GWE and GWI seem to be accurate non-invasive echocardiographic parameters of myocardial function to predict early LV remodeling and adverse outcomes after an AMI. They should be used to optimize medical treatment and compliance, correct modifiable cardiovascular risk factors, and investigate for residual myocardial ischemia.

### Study Limitations

Our study has several limitations. The research was conducted in only one interventional cardiology center, and the sample size of STEMI patients was relatively small due to the inclusion criteria. When assessing myocardial work through pressure-strain loops, we calculated LV pressure using cuff sphygmomanometer data. However, it’s important to note that these non-invasive measurements have known limitations that should be taken into consideration. This method involves the measurement of systolic blood pressure, which serves as an estimation of LV pressure, under the assumption that there is no LV outflow obstruction. Patients having a poor echocardiographic window during the index admission were excluded, which may have caused bias in the selection process. Cardiac magnetic resonance imaging or single-photon emission computed tomography myocardial perfusion was not accessible for measuring the infarct dimension. More research is required to investigate the predictive value of non-invasive myocardial indices in STEMI patients.

## 5. Conclusions

Non-invasive myocardial work indices represent a repeatable and accurate examination method to measure LV performance. Among them, GWI and GWE may be the most suitable predictors of early LV remodeling and MACEs in STEMI patients with preserved LVEF following an efficient primary PCI.

## Figures and Tables

**Table 1:** Characteristics of STEMI patients split for LV remodeling at 3 months.

Parameter	Group A No LV Remodeling (*n* = 188)	Group B LV Remodeling (*n* = 58)	All STEMI Patients with LVEF ≥ 50% Following PCI (*n* = 246)	*p* Value
At baseline				
Age (years)	64.8 ± 12	72.0 ± 13	66 ± 13	**<0.0001**
Male sex (*n*, %)	130 (70)	48 (82)	178 (73)	0.06
Diabetes mellitus	104 (56)	34 (58)	138 (56)	0.78
Systemic hypertension	117 (63)	54 (94)	171 (70)	**<0.0001**
Hypercholesterolemia	140 (74)	49 (85)	189 (76)	0.05
Smoking history	66 (35)	29 (51)	95 (39)	**0.03**
Obesity	51 (27)	11 (19)	62 (25)	0.22
SBP (mmHg)	129.2 ± 13.2	144.6 ± 13.5	130.8 ± 12.5	**<0.0001**
DBP (mmHg)	78.5 ± 6.2	86.5 ± 18.1	73.7 ± 12.7	**<0.0001**
Heart rate (beats/min)	81 ± 19	80 ± 21	80 ± 20	0.73
Chronic renal failure	49 (26)	16 (28)	65 (26)	0.11
Killip class I (%) II (%) III (%) IV (%)	51 (27) 109 (58) 18 (10) 10 (5)	1 (2) 11 (19) 29 (50) 17 (29)	52 (21) 120 (48) 47 (19) 27 (12)	**<0.0001** **<0.0001** **<0.0001** **<0.0001**
Peak CPK-MB (IU/L), median (25th, 75th percentile)	220 (87.2, 449.7)	317 (200.0, 620.0)	251 (107.7, 449.2)	**<0.01**
NT-proBNP (ng/L) median (25th, 75th percentile)	227 (58, 310)	921 (214, 2550)	**228 (83, 520)**	0.68
eGFR (mL/min/1.73m^2^)	75.8 ± 21.7	73.8 ± 23.0	79.4±	
Culprit vessel LAD (%) LCX (%) RCA (%) Initial patency of IRA	95 (51) 22 (12) 69 (37) 37 (20)	22 (38) 8 (14) 24 (42%) 7 (12)	97 (40) 30 (12) 93 (41) 44 (18)	0.07 0.68 0.12 0.16
Coronary artery disease 1-vessel (%) 2-vessel (%) 3-vessel (%)	126 (68) 32 (17) 16 (15)	13 (22) 19 (33) 26 (45)	139 (62) 51 (23) 42 (19)	**<0.0001** **<0.01** **<0.0001**
Medication at discharge ACEI or ARB Beta-blocker Calcium antagonists Statins	151 (81) 147 (79) 41 (22) 138 (74)	45 (78) 44 (76) 14 (24) 42 (72)	196 (80) 191 (78) 55 (23) 180 (74)	0.60 0.62 0.74 0.75
Major cardiac events during the 4-year follow-up				
Hospitalizations for HF *n* (%)	17 (9)	11 (19)	29 (12)	**0.03**
Repeated PCI *n* (%)	8 (4)	7 (12)	15 (6)	**0.02**
CABG *n* (%)	3 (2)	2 (3)	5 (2)	0.27
Sudden cardiac deaths *n* (%)	0 (0)	0 (0)	0 (0)	1.00
Total cardiac events *n* (%)	28 (15%)	20 (34%)	48 (19%)	**0.01**

Notes: Statistically significant values are shown in bold (*p* < 0.05). Continuous variables that are normally distributed are presented as mean ± 1 standard deviation; continuous variables that are not normally distributed are presented as median (25th, 75th percentile). Abbreviations: ACEI, angiotensin-converting enzyme inhibitor; ARB, angiotensin receptor blocker; BNP, brain natriuretic peptide; CABG, coronary artery by-pass graft; CPK-MB, creatine kinase MB isoenzymes; DBP, diastolic blood pressure; eGFR, estimated glomerular filtration rate; IRA, infarct-related artery; IU, international units; HF, heart failure; LAD, left anterior descending artery; LCX, left circumflex artery; LVEF, left ventricular ejection fraction; PCI, percutaneous coronary intervention; RCA, right coronary artery; SBP, systolic blood pressure; STEMI, ST-segment elevation acute myocardial infarction.

**Table 2:** Echocardiographic Findings.

Parameter	Group A without LV Remodeling *n* = 188	Group B with LV Remodeling *n* = 58	All STEMI Patients *n* = 246	*p* Value
Baseline				
LVEF (%)	57.5 ± 7.1	56.3 ± 4.5	57.1 ± 6.1	0.22
LVEDV (mL)	106 ± 15	104 ± 12	105 ± 15	0.35
LVESV (mL)	44 ± 7.5	42 ± 8.6	43.5 ± 9.1	0.08
Stroke volume index (mL/m^2^)	41 ± 10.4	39.6 ± 11.4	40.3 ± 10.8	0.37
E/A ratio	1.09 ± 0.32	1.02 ± 0.28	1.07 ± 0.30	0.13
WMSI	2.23 ± 0.19	2.25 ± 0.23	2.23 ± 0.21	0.09
GLS (%)	−18.3 ± 3.5	−17.6 ± 3.9	−18.0 ± 3.6	0.19
GWI (mmHg%)	10,886 + 465	1529 ± 142	1802 ± 439	**<0.0001**
GWE (%)	89.6 ± 6.6	77.7 ± 4.3	87.2 ± 7.1	**<0.0001**
GWW (mmHg%)	198.3 ± 47	224 ± 21	204.3 ± 52.8	**0.001**
GCW (mmHg%)	2110 ± 195	1622 ± 148	1994.40 ± 278	**<0.0001**
After 3 months				
LVEF (%)	61.3 ± 7.2	56.7 ± 8.2	59.1 ± 7.6	**<0.0001**
LVEDV (mL)	123 ± 27	132 ± 30	127.5 ± 28	**0.03**
LVESV (mL)	47 ± 14	52 ± 12	50 ± 13	**0.01**
Stroke volume index (mL/m^2^)	46.2 ± 5.5	43.5 ± 7.5	45.85 ± 6.5	**<0.01**
E/A ratio	1.08 ± 0.30	1.01 ± 0.24	1.06 ± 0.31	0.10
WMSI	1.95 ± 0.3	2.14 ± 0.5	2.03 ± 0.3	**<0.001**
GLS (%)	−20.1 ± 2.8	−19.2 ± 3.1	−19.8 ± 2.9	**0.03**
GWI (mmHg%)	1938 ± 151	1868 ± 236	1921 ± 177	**<0.001**
GWE (%)	91.3 ± 4.3	83.6 + 5.8	88.9 ± 5.7	**<0.001**
GWW (mmHg%)	179.9 ± 55	209.0 ± 64	95.5 ± 68	**<0.001**
GCW (mmHg%)	2206 ± 235	2080 ± 302	1656 ± 288	**0.001**

Notes: Statistically significant values are shown in bold (*p* < 0.05). Values are presented as mean ± 1 standard deviation. Abbreviations: A, late diastolic wave velocity; E, early diastolic wave velocity; GCW, global constructive work; GLS, global longitudinal strain; GWE, global work efficiency; GWI, global work index; GWW, global wasted work; LVEF, left ventricular ejection fraction; LVEDV, left ventricular end-diastolic volume; LVESV, left ventricular end-systolic volume; STEMI, ST-segment elevation acute myocardial infarction; WMSI, wall motion score index.

**Table 3:** Predictors for LV remodeling in STEMI patients with LVEF ≥ 50% after successful primary PCI.

Univariate Logistic Regression	Odds Ratio	95% CI	*p* Value
Age (years)	1.05	1.03–1.07	**<0.01**
Systemic hypertension	3.72	1.35–9.62	**<0.01**
Hypercholesterolemia	3.4	1.50–9.09	**<0.01**
Smoking	0.5	0.28–0.83	**<0.01**
Killip class	3.98	1.65–9.32	**<0.001**
Peak CPK-MB (IU/L)	1.24	1.07–1.85	**<0.0001**
2-vessel CAD	2.4	1.16–4.28	**<0.02**
3-vessel CAD	3.8	1.80–7.34	**<0.0001**
Baseline GWI (mmHg%)	3.68	2.53–3.35	**<0.0001**
Baseline GCW (mmHg%)	2.94	3.17–4.18	**<0.0001**
Baseline GWE (%)	0.72	0.66–0.79	**0.01**
Baseline GWW (mmHg%)	1.01	1.00–1.02	**0.0001**
Multivariate logistic regression	Odds Ratio	95% CI	*p*-value
Killip class	2.44	1.18–5.01	**<0.001**
Baseline GWI (mmHg%)	0.96	0.94–0.98	**<0.001**
Baseline GWE (%)	0.56	0.40–0.78	**<0.001**

Notes: Statistically significant values are highlighted in bold (*p* < 0.05). Values are presented as mean ± 1 standard deviation. Abbreviations: CAD, coronary artery disease; CPK-MB, creatine phosphokinase kinase MB isoenzymes; GCW, global constructive work; GWE, global work efficiency; IU, international units; GWI, global myocardial work index; GWW, global wasted work. STEMI, ST-segment elevation acute myocardial infarction.

**Table 4:** Predictors for MACEs during the 4-year follow-up after a STEMI.

	Univariate Logistic Regression			Multivariate Logistic Regression		
	Odds Ratio	095% CI	*p* Value	Odds Ratio	95% CI	*p* Value
Killip class	2.75	1.89–3.98	**<0.0001**	1.89	1.05–3.3	**<0.001**
3-vessel CAD	2.4	1.22–4.80	**<0.012**	-	-	-
NT-pro BNP (ng/L)	1.00	1.00–1.00	**<0.01**	-	-	-
CK-MB (IU/L)	1.00	1.00–1.00	**<0.01**	1.00	1.00–1.00	**<0.01**
GWI	0.99	0.99–0.99	**<0.0001**	0.99	0.98–0.99	**<0.0001**
GWE	0.81	0.76–0.86	**<0.0001**	0.85	0.79–0.92	**<0.001**

Notes: Statistically significant values are highlighted in bold (*p* < 0.05). Abbreviations: MACEs, major cardiac events; STEMI, ST-segment elevation acute myocardial infarction; CAD, coronary artery disease; CPK-MB, creatine phosphokinase kinase MB isoenzymes; IU, international units; CAD, coronary artery disease; GWI, global myocardial work index; GCW, global constructive work.

## Data Availability

Not applicable.
